# Transcriptomic Analysis of Resistant and Susceptible* Bombyx mori* Strains Following BmNPV Infection Provides Insights into the Antiviral Mechanisms

**DOI:** 10.1155/2016/2086346

**Published:** 2016-04-18

**Authors:** Gang Li, Heying Qian, Xufang Luo, Pingzhen Xu, Jianhua Yang, Mingzhu Liu, Anying Xu

**Affiliations:** Sericultural Research Institute, Jiangsu University of Science and Technology, Zhenjiang, Jiangsu 212018, China

## Abstract

*Purpose*. To decipher transcriptomic changes and related genes with potential functions against* Bombyx mori* nucleopolyhedrovirus infection and to increase the understanding of the enhanced virus resistance of silkworm on the transcriptomic level.* Methods*. We assembled and annotated transcriptomes of the Qiufeng (susceptible to infection) and QiufengN (resistant to infection) strains and performed comparative analysis in order to decipher transcriptomic changes and related genes with potential functions against BmNPV infection.* Results*. A total of 78,408 SNPs were identified in the Qiufeng strain of silkworm and 56,786 SNPs were identified in QiufengN strain. Besides, novel AS events were found in these 2 strains. In addition, 1,728 DEGs were identified in the QiufengN strain compared with Qiufeng strain. These DEGs were involved in GO terms related to membrane, metabolism, binding and catalytic activity, cellular processes, and organismal systems. The highest levels of gene representation were found in oxidative phosphorylation, phagosome, TCA cycle, arginine and proline metabolism, and pyruvate metabolism. Additionally, COG analysis indicated that DEGs were involved in “amino acid transport and metabolism” and “carbohydrate transport and metabolism.”* Conclusion*. We identified a series of major pathological changes in silkworm following infection and several functions were related to the antiviral mechanisms of silkworm.

## 1. Introduction

The domesticated silkworm,* Bombyx mori*, which has been reared by humans for more than 5000 years, is an agriculturally important insect and has economically important values in sericulture [1]. In addition, the silkworm is a key model of the Lepidoptera, a group of holometabolous insects [2].* Bombyx mori* nucleopolyhedrovirus (BmNPV) is an exclusive pathogen of the silkworm, frequently causing serious economic damage to sericulture production [3]. Nowadays, no therapeutic agents are available to effectively control BmNPV infection.

The infection cycle of BmNPV is mediated by two phenotypically different viral phenotypes: the occlusion-derived virus and the second virion phenotype, the budded virus [4]. The genome of BmNPV has been completely sequenced [5] and the pathogenesis of systemic BmNPV infection has been largely investigated. Evidence has shown that NPV infection typically results in a global shutoff of insect gene expression, and protein synthesis in host cells begins at about 12–18 hours after infection [6]. Katsuma et al. had demonstrated that the activation of mitogen-activated protein kinases (MAPK) signaling pathways was required for efficient infection by BmNPV [7]. Recently, a major emphasis in silkworm research has been given to develop NPV resistant varieties by transgenesis [8]. However, studies on antiviral mechanism in insects are still in their infancy, and the defense mechanisms at an early stage of viral infection need to be investigated.

Recently, RNA-sequencing (RNA-seq), based on deep sequencing technology, is a powerful tool for transcriptome analyses [9]. RNA-seq has revealed novel genes and transcripts, tissue-specific alternative splicing, and genomic structural variations [10, 11]. In recent years, RNA-seq has been employed to investigate the molecular mechanisms of numerous physiological events in the silkworm [12, 13]. For instance, Wang et al. performed transcriptome analysis of the brain of the silkworm* Bombyx mori* infected with BmNPV and revealed that differentially expressed genes (DEGs) involved in synaptic transmission, circadian rhythms, and the serotonin receptor signaling pathway of* Bombyx mori* were related to the locomotor activity of* Bombyx mori* following BmNPV infection [14].

In the current study, the fat bodies from two silkworm strains of* Bombyx mori*, Qiufeng and QiufengN, were used for identifying the complexity of the antiviral mechanism in silkworm. The Qiufeng strain is commercially available and susceptible to BmNPV infection [15]. The QiufengN strain is identified to be highly resistant to BmNPV infection in our previous study (data not shown). Besides, RNA-seq technology was applied to analyze the transcription profile between the Qiufeng strain and QiufengN strain following BmNPV infection. The purpose of this study was to decipher transcriptomic changes and related genes with potential functions against BmNPV infection and to increase the understanding of the enhanced virus resistance of silkworm on the transcriptomic level.

## 2. Materials and Methods

### 2.1. Viruses and Silkworm Strain Samples

The silkworm* Bombyx mori* strains, Qiufeng and QiufengN (a disease-resistant variety), were maintained in our laboratory and reared using fresh mulberry leaves under laboratory conditions at 27 ± 1°C with 70–85% relative humidity and a photoperiod of 12 h light/12 h dark. BmNPV was stored in our laboratory. The fifth instar larvae were harvested after the 4th ecdysis and were used for the experiments.

### 2.2. Oral Infection of the Silkworms with BmNPV for Transcriptome Analyses

Fifty newly molted silkworm larvae of the 5th instar from each of the strains (Qiufeng and QiufengN) were deprived of food 2 h before infection. For infection, the silkworms were orally inoculated with 7 *μ*L of BmNPV suspension (2 × 10^9^ polyhedra/mL). The fat bodies of these 2 strains were collected at 48 h after infection, respectively. Specially, the samples from Qiufeng and QiufengN in this study were named as T01 and T02, respectively. The samples were isolated on ice and placed immediately in liquid nitrogen and frozen at −70°C until use.

### 2.3. RNA Extraction

Total RNA was extracted from each sample according to the manufacturer's instructions using TRIzol LS (Invitrogen, CA, USA), as previously described [16]. The RNA was then treated with RNase-free DNase I in order to remove genomic DNA contamination. The purity and quality of the RNA were determined by assessing the absorbance ratio OD260/280 (1.9–2.0) using NanoDrop ND-1000 Spectrophotometer (NanoDrop, Wilmington, DE). Besides, the RNA was quantified with a Qubit 2.0 Fluorometer (Invitrogen Corporation, Carlsbad, CA, USA) in accordance with the manufacturer's instructions. The integrity of the RNA samples was verified using an Agilent 2100 Bioanalyzer (Agilent, Santa Clara, CA, USA) with RNA Integrity Number (RIN) values over 8.0.

### 2.4. cDNA Library Construction and Illumina RNA-seq

Libraries construction and the RNA-seq were carried out by the Biomarker Biotechnology Corporation (Beijing, China). Briefly, the poly(A) mRNAs were enriched from total RNA with oligo(dT) magnetic beads and then the enriched mRNAs were fragmented by using fragmentation buffer. Using these cleaved mRNA fragments as templates, the first strand of cDNA was synthesized with random hexamer primers. The second cDNA strand was then synthesized with buffer, RNase H, dNTPs, and DNA polymerase I. Thereafter, the double-stranded cDNA fragments were purified with Agencount AMPure XP beads (Beckman Coulter, Brea, CA, USA) and resolved with elution buffer (EB) for end repair and the addition of poly(A). Then, the repaired short fragments were ligated with sequencing adaptors. The suitable size of fragments was selected and purified by AMPure XP beads (Beckman Coulter, Brea, CA, USA), and then the purified cDNA templates were enriched through PCR amplification. After the library was constructed, the Agilent 2100 Bioanalyzer (Agilent, Santa Clara, CA, USA) and the Qubit 2.0 Fluorometer (Invitrogen Corporation, Carlsbad, CA, USA) were used to calculate the molar concentration and confirm the insert size. The cDNA libraries were sequenced on an Illumina HiSeq 2500 instrument (Illumina Inc., San Diego, CA, USA) that generated paired-end reads of 125-bp.

### 2.5. Sequencing Data Processing and Assembly

After removing the adaptor sequences and those reads with unknown nucleotides of more than 5% (N bases) or those low-quality reads (more than 50% of low-quality bases with* Q*-value ≤5 in a single read), the clean reads were obtained after being filtered from the raw reads. The cleaned RNA-seq reads were then mapped to the silkworm genome sequence derived from the SilkDB database (http://silkworm.swu.edu.cn/silkdb/) using the software TopHat2 [17]. The transcript levels were quantified using the fragments per kilobase of exon per million fragments mapped (FPKM) [18]. A threshold of FPKM ≥ 0.1 was set to ensure that the genes were expressed in these two groups.

### 2.6. SNP Identification and Analysis of Alternative Splicing (AS) Events

Based on the alignment results of cleaned RNA-seq reads being mapped to the reference sequence using the software TopHat2 [17], SAMtools software [18] was applied to perform single nucleotide polymorphism (SNP) calling. The filtering threshold for high quality SNPs was set as follows: (1) the alignment score of TopHat2 was no less than 50; (2) the interval of called SNPs was more than 5 bases; (3) the quality score was no less than 2; (4) the sequencing depth was ≥ 5x and ≤ 100x. Furthermore, the SNPs of the two strains were compared. Total number of SNPs and heterozygous SNPs were calculated. The ratio of transition and transversion was calculated via analyzing each type of DNA substitution. Besides, the SNP density was calculated in these 2 groups.

The AS events contain several typical types, including alternative 5′ splice site, alternative 3′ splice site, intron retention (IR), exon skipping (ES), alternative last exon, alternative first exon, and mutually exclusive exons [10]. In this study, the Cufflinks software [19] was employed to annotate all novel AS events that occur in QiufengN and Qiufeng strain based on the intron-containing mapped data.

### 2.7. De Novo Assembly and Functional Annotation

Transcriptome de novo assembly was performed on the mapped data using Cufflinks software [19]. The new genes [20] were identified via discarding the sequences encoding less than 50 amino acids and the sequence containing only one intron. The assembled unigenes were annotated based on sequence comparability with previously identified genes with descriptions. For assignments of the predicted gene descriptions, a BLASTx alignment [20] (*E* value < 1.00*E* − 05) was carried out between our assembled unigenes and the protein databases such as NCBI nonredundant protein (NR) database (http://www.ncbi.nlm.nih.gov/), the Kyoto Encyclopedia of Genes and Genomes (KEGG) pathway database (http://www.genome.jp/kegg/), Swiss-Prot protein database (http://web.expasy.org/docs/swiss-prot_guideline.html), and the Cluster of Orthologous Groups (COG) database (http://www.ncbi.nlm.nih.gov/COG/). The best aligning results that had the highest sequence similarity with our unigenes were used to decide the sequence direction of unigenes and determine their functional annotations.

### 2.8. Differential Expression and Functional Analysis of Differentially Expressed Genes (DEGs)

Differential gene expression analysis was carried out to compare the QiufengN and Qiufeng libraries using EBseq [17] which is developed with empirical Bayesian approach in RNA-seq data comparing two or more biological conditions [17]. The false discovery rate (FDR) [18] was applied to determine the *P* value thresholds via Benjamini-Hochberg method [21]. The fold change for each transcript between these 2 samples was calculated as the ratio of the FPKM values. In this study, the criteria of a fold change (FC) ≥ 2 and a FDR < 0.01 were used to identify the DEGs. Heat maps of the significant genes were generated with the heatmap.2 package [10] in R. Besides, a BLASTx alignment [20] (*E* value < 1.00*E* − 05) was performed between the identified DEGs and the protein databases like NR database (http://www.ncbi.nlm.nih.gov/), KEGG pathway database (http://www.genome.jp/kegg/), Swiss-Prot protein database (http://web.expasy.org/docs/swiss-prot_guideline.html), and the COG database (http://www.ncbi.nlm.nih.gov/COG/).

Moreover, GO and KEGG pathway enrichment analyses for these DEGs were conducted using hypergeometric tests with the assembled reference transcriptome set as the background. For the enrichment analyses, a *P* value of < 0.05 was chosen as the threshold to determine the significant enrichment. Besides, the tool of topGO [22] was applied to improve the specificity and discovery sensitivity by considering the interrelationships of the identified GO terms. Additionally, the sequences of DEGs were subjected to the COG database (http://www.ncbi.nlm.nih.gov/COG/) and the corresponding COG annotation results were extracted.

## 3. Results

### 3.1. General Transcriptional Patterns

**Figure 1:**
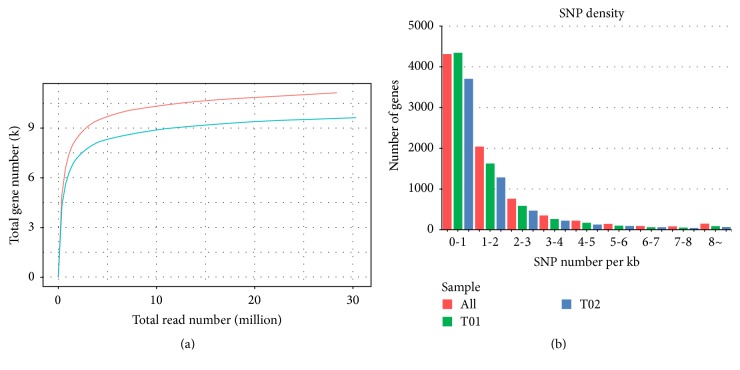
Saturation curve of transcriptomic sequencing reads for samples and single nucleotide polymorphism (SNP) density distribution. (a) Saturation curve of transcriptomic sequencing reads for samples of the silkworm strains QiufengN and Qiufeng. Red line, samples of the Qiufeng strain; green line, samples of the QiufengN strain. (b) SNP density distribution of the QiufengN and Qiufeng strain. T01 indicates the sample of Qiufeng strain. T02 indicates the sample of QiufengN strain.

To understand and monitor the changes in gene expression in silkworm disease-resistant strain (QiufengN), fat bodies from BmNPV-infected QiufengN and BmNPV-infected Qiufeng were used to build 2 libraries for transcriptome sequencing. After filtering, a total of 14.78 Gb clean data (more than 7.15 Gb for T01 and T02, resp.) were obtained from the two libraries (Table 1). The quality score (*Q*-score) of > 30 (*Q*30) and guanine-cytosine (GC) percentages of the two groups were 88.00% and 45.41% and 88.81% and 46.41%, respectively (Table 1). Besides, the ratios of the unique mapped reads that accounted for the mapped reads in these two groups (T01 and T02) were 68.53% and 76.29%, respectively. In addition, the results showed that the present sequencing data were completely saturated and were adequate for the follow-up analysis (Figure 1(a)).

### 3.2. SNP Calling and Alternative Splicing Analysis

SNPs were discovered by mapping filtered reads to the reference sequences for further application of the RNA-seq data. As summarized in Table 2, a total of 78,408 SNPs were identified in the Qiufeng strain of silkworm after quality control and filtration, including 44,121 genic SNPs and 34,287 intergenic SNPs. On the other hand, a total of 56,786 SNPs were identified in QiufengN strain, consisting of 34,858 genic SNPs and 21,928 intergenic SNPs. The proportions of transition substitutions were 63.22% compared with a smaller proportion (36.78%) of transversion with regard to the Qiufeng strain. While the proportion of transition substitutions was 64.46%, the proportion of transversion substitutions was 35.54% in the QiufengN strain. Furthermore, 15.28% SNPs were heterozygous in the Qiufeng strain and 23.56% were heterozygous in the QiufengN strain. The mean number of SNPs per kilobase in the ORF region for the two strains was shown in Figure 1(b).

To identify the differences in novel AS events ratios between the QiufengN and Qiufeng strains, the Cufflinks software was applied to calculate the changes in the relative splice abundances. In all, we found that a total of 7,874 novel simple AS events that fell into six different categories were identified in QiufengN strain, including 3,107 ES events, 481 IR events, 625 alternative 5′ splice site events, 749 alternative 3′ splice site events, 860 alternative last exon events, and 2,052 alternative first exon events. On the other hand, total 5937 novel AS events that fell into these six different categories were identified in Qiufeng strain, including 2,257 ES events, 313 IR events, 504 alternative 5′ splice site events, 602 alternative 3′ splice site events, 705 alternative last exon events, and 1,556 alternative first exon events. We found that, in these two strains, the use of alternative 5′ splice sites is a little more frequent than the use of alternative 3′ splice sites. In addition, our mapped data showed that ES and alternative first exon were common events compared with other AS events in these two strains.

### 3.3. Functional Annotation by Searching against Public Databases

Approximately 1200 unique sequences were identified. For validation and annotation of these genes, sequence similarity search was carried out based on BLASTx (cut-off *E* value 10^−5^) searches against 5 public databases: NR database, GO database, KEGG database, Swiss-Prot protein database, and COG database. The results indicated that, out of 1200 unique sequences, 1053 could be annotated with known proteins in NR database as reference. In addition, 530 unigenes had BLAST hits in Swiss-Prot database. A total of 527 unique best BLASTx matches were produced from GO database. The unigenes obtaining gene descriptions from KEGG database were 255. In all, 1053 of the assembled unigene sequences showed homology to available known sequences, while unigenes without hits (147, 12.20%) might represent new genes or errors in the assembly procedure.

### 3.4. Identification of DEGs between QiufengN and Qiufeng Strains

**Figure 2:**
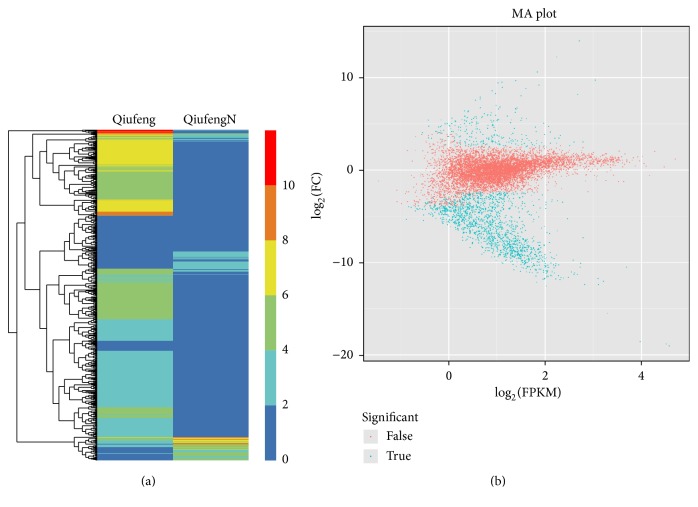
Identification of differentially expressed genes (DEGs). (a) Heat map of DEGs between QiufengN and Qiufeng strains. Columns indicate different samples. Rows represent different DEGs. Different colors indicate the gene expression levels in the samples that measured as log_2_ (FPKM + 1). (b) MA plot of DEGs. Red points represent genes without differential expression. Green points indicate DEGs. FC, fold change. FPKM, fragments per kilobase of transcript.

The difference in levels of DEGs between QiufengN and Qiufeng strains was calculated according to RPKM value of the obtained unigenes. Under the criteria of FC ≥ 2 and FDR < 0.01, a total of 1,728 DEGs were identified in QiufengN sample compared with Qiufeng sample, of which 242 genes were upregulated and 1,540 were downregulated. The heat map and MA plot of DEGs were shown in Figures 2(a) and 2(b). These results demonstrated the overall difference in transcriptional expression level of DEGs between QiufengN and Qiufeng strains. In addition, a total of 1,706 DEGs could be annotated based on searches against 5 public databases: NR, GO, KEGG, Swiss-Prot protein database, and COG database. Thereinto, 458, 903, 366, 1,006, and 1,706 DEGs could be annotated with known proteins in COG, GO, KEGG, Swiss-Prot, and NR database as reference, respectively.

### 3.5. GO and KEGG Enrichment Analysis of the DEGs

**Figure 3:**
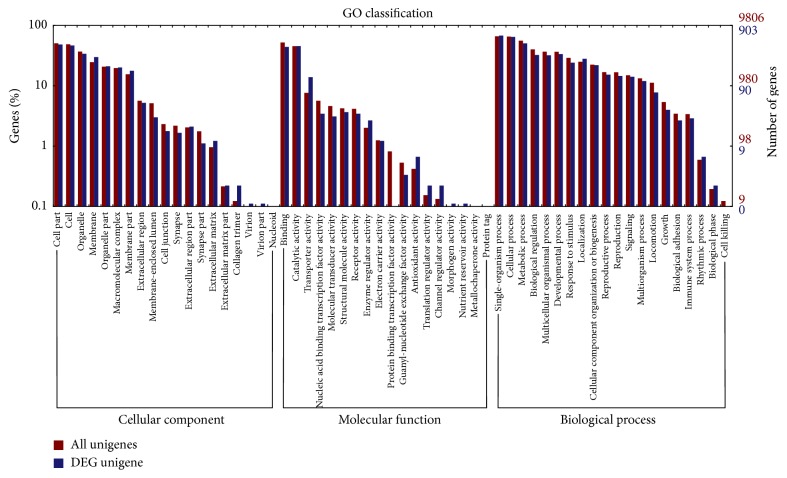
Gene Ontology (GO) classification of DEGs.

The DEGs were assigned to different GO categories in terms of cellular component (CC), molecular function (MF), and biological process (BP) to determine their functional classifications (Figure 3). GO functional annotation of the 1,728 DEGs indicated that 724 DEGs were enriched in MF ontology, 590 DEGs were enriched in CC ontology, and 734 DEGs were enriched in BP ontology. In general, 19, 18, and 20 catalogs of CC, MF, and BP were clustered, respectively. The majority of the identified DEGs were assigned to the cell, organelle, and membrane in the CC category. Besides, the results showed that the DEGs were significantly concerned with binding and catalytic activity in the MF category. Additionally, we found that, in the category of BP, the majority of the DEGs were significantly involved in single-organism process, cellular process, and metabolic process. In addition, the results of topGO tool analysis showed that the DEGs were significantly related with structural constituent of pupal chitin-based cuticle, disaccharide transport, and membrane (Table 3).

**Figure 4:**
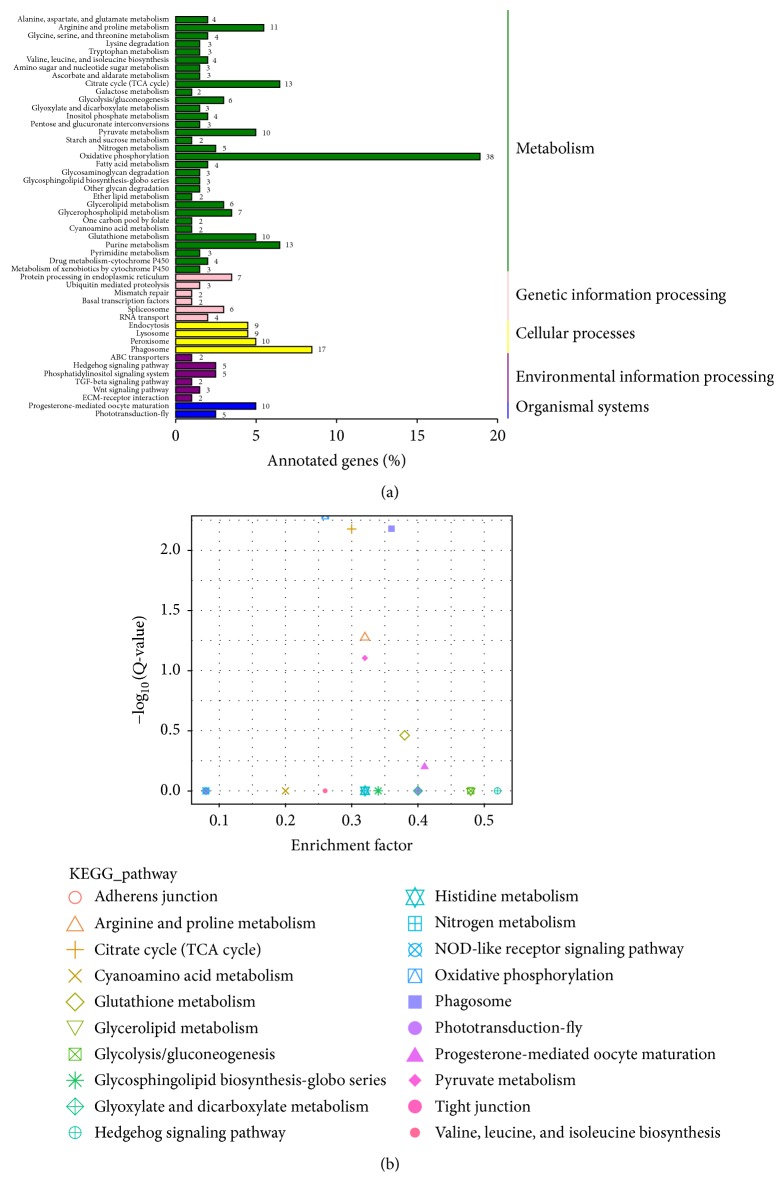
KEGG enrichment analysis of DEGs. (a) Histogram representation of KEGG pathways of DEGs. *x*-axis represents the number and percentage of DEGs enriched in a specific pathway. *y*-axis indicates the KEGG pathways. (b) Scatter diagram of KEGG pathways. The *x*-axis “enrichment factor” indicates the ratio of the proportion of all genes annotated to a pathway to the proportion of the DEGs annotated to a pathway. Less enrichment factor indicates more significance of the DEGs being annotated to the pathway. *Q*-value represents corrected *P* value. Thus, the point near the top left corner in the figure had more significance.

On the other hand, the results of KEGG enrichment analysis indicated that a total of 201 annotated DEGs were assigned into 97 pathways. As shown in Figure 4(a), the pathways were mainly classified into 5 categories, namely, metabolism, genetic information processing, cellular processes, environmental information processing, and organismal systems. Besides, we found that the most representative pathway related to metabolism was oxidative phosphorylation (Figure 4(a)). On the other hand, Figure 4(b) showed that the oxidative phosphorylation, phagosome, citrate cycle (TCA cycle), arginine and proline metabolism, and pyruvate metabolism pathways were most significantly enriched.

### 3.6. COG Analysis of the DEGs

**Figure 5:**
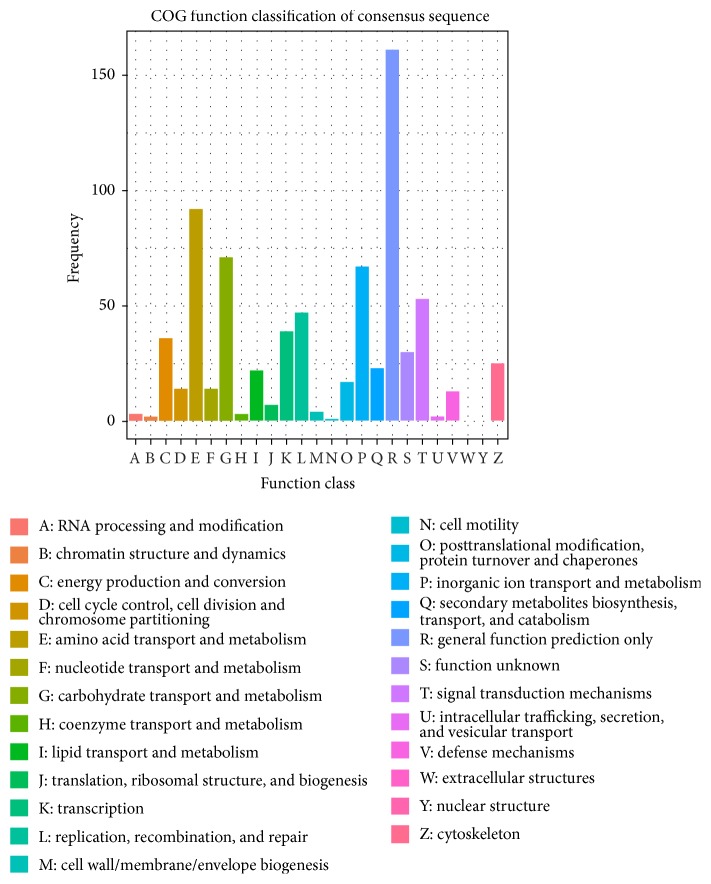
Histogram representation of clusters of orthologous groups (COG) classification of DEGs. The *y*-axis “Frequency” indicates the number of genes in a specific function cluster. The figure keys show a description of the 25 function categories that were functionally classified by genes.

To further analyze the completeness of the transcriptome library and evaluate the effectiveness of annotations, COG analysis of the DEGs was carried out, which could provide more information about the potential functions of genes. The results indicated that the DEGs were classified into 25 functional categories (Figure 5). Among those categories, the cluster of “general function prediction only” was the largest group, followed by “amino acid transport and metabolism” and “carbohydrate transport and metabolism.” Cluster for “cell motility” was the smallest group. Other moderate groups such as signal transduction mechanisms were also identified.

## 4. Discussion

The resistance mechanisms of* Bombyx mori* against BmNPV remain obscure. In the present study, RNA-seq technology was applied to profile the transcriptome of QiufengN as well as Qiufeng strains and approximately 14.78 Gb clean data (more than 7.15 Gb for QiufengN and Qiufeng, resp.) were obtained from the two libraries. A total of 78,408 SNPs were identified in the Qiufeng strain of silkworm and 56,786 SNPs were identified in QiufengN strain. Besides, novel AS events were found in these 2 strains. In addition, 1,728 DEGs (242 upregulated and 1,540 downregulated) were identified in the QiufengN strain compared with the samples of Qiufeng strain. Analyses of GO categories revealed that these DEGs were mainly involved in GO terms related to membrane, metabolism, binding and catalytic activity, cellular processes, and organismal systems, such as oxidative phosphorylation and TCA cycle. Pathway analysis with KEGG annotation showed that the highest levels of gene representation were found in oxidative phosphorylation, phagosome, TCA cycle, arginine and proline metabolism, and pyruvate metabolism. Additionally, COG analysis indicated that DEGs were associated with “amino acid transport and metabolism” and “signal transduction mechanisms.”

In this study, we identified a total of 78,408 SNPs in the Qiufeng strain of silkworm after quality control and filtration, including 44,121 genic SNPs and 34,287 intergenic SNPs. By contrast, 56,786 SNPs were identified in QiufengN strain, consisting of 34,858 genic SNPs and 21,928 intergenic SNPs. These results indicated that the complex regulatory machinery was involved in* Bombyx mori *following BmNPV infection. On the other hand, 1,728 DEGs were identified in the QiufengN strain (resistant to infection) compared with the samples of Qiufeng strain (susceptible to infection), indicating that a relatively large number of genes were involved in antiviral mechanisms in silkworm.

Oxidative stress results from free radicals such as reactive oxygen species (ROS) [23]. Small quantities of ROS are spontaneously formed as byproducts of redox processes under normal conditions such as oxidative phosphorylation in the mitochondria [24]. The findings of Gaikwad et al. demonstrated that oxidative stress was increased due to D-galactose in D-galactose treated larvae of silkworm [25]. Besides, evidence has shown that cellular organelles which are surrounded with lipid membranes could be damaged due to lipid peroxidation, a result of oxidative stress [26]. In this study, we found that DEGs identified between Qiufeng strain and QiufengN strain were concerned with various GO terms and pathways such as organelle and membrane, and oxidative phosphorylation, which indicated that oxidative stress might be related to the defensive function of silkworm following BmNPV infection. On the other hand, multiple metabolism-related functions were enriched by DEGs identified in this study, including pyruvate metabolism, TCA cycle, and arginine and proline metabolism. Xu et al. revealed that many metabolic activities of the midgut like carbohydrate metabolism, the TCA cycle, and oxidative phosphorylation were probably affected during the wandering stage of* Bombyx mori* silkworm [27]. In line with the previous study, we suggested that many metabolic activities such as oxidative phosphorylation were affected and had biological relevance for the antiviral mechanisms of silkworm. Further experiments are required to test this finding.

Moreover, DEGs were identified to be related with the environmental information processing, such as Phosphatidylinositol signaling pathway. DEGs related to this pathway were as follows: BGIBMGA002122; BGIBMGA003299; BGIBMGA007232; BGIBMGA012694; BGIBMGA012695. Ample evidence has shown that Phosphatidylinositol signaling pathway is significantly related to immune response [28, 29]. In addition, several immune-responsive genes such as serpin-5 were probably involved in the host's antiviral mechanisms of* Bombyx mori* following infection [6]. In this study, COG analysis also revealed DEGs related signal transduction mechanisms. Collectively, understanding the physiological functions of these DEGs involved in signaling pathway will require further characterization in order to determine their roles in immunity or other processes. However, experiments are needed to verify this result.

## 5. Conclusions

We identified that a rather larger number of DEGs were involved in some functional categories, indicating a series of major pathological changes in the silkworm following infection and several functions were related to the antiviral mechanisms of silkworm. Oxidative stress might be related to the defensive function of silkworm following BmNPV infection. In addition, many metabolic activities such as oxidative phosphorylation were affected and had biological relevance for the antiviral mechanisms of silkworm. Moreover, understanding the physiological functions of these DEGs involved in signaling pathway will require further characterization in order to determine their roles in immunity or other processes. These findings provide new research directions that may deepen our understanding of virus resistance of silkworm on the molecular level. Because there are some false positive rates in RNA-seq data, further experimental verifications like real-time PCR are needed.

## Figures and Tables

**Table 1:** Summary statistics of the transcriptome sequence data in the two libraries.

		T01	T02
Clean reads (pair-end)	Read number	28,402,151	30,308,707
	Base number	7,151,259,373	7,632,852,036
	GC content	45.41%	46.41%
	% ≥ *Q*30	88.00%	88.81%

Mapping to genome (single-end)	Total reads	56,804,302	60,617,414
	Mapped reads	41,047,811	47,751,117
	Mapped ratio	72.26%	78.77%
	Unique mapped reads	38,826,519	46,243,843
	Unique mapped ratio	68.35%	76.29%

T01 indicates the sample of BmNPV-infected Qiufeng strain. T02 indicates the sample of BmNPV-infected QiufengN strain.

**Table 2:** Statistics of SNPs discovered from RNA-seq data of QiufengN and Qiufeng strains of silkworm.

Sample	SNP number	Genic SNP	Intergenic SNP	Transition	Transversion	Heterozygosity
Qiufeng	78,408	44,121	34,287	63.22%	36.78%	15.28%
QiufengN	56,786	34,858	21,928	64.46%	35.54%	23.56%

SNP: single nucleotide polymorphism.

**Table 3:** The top 5 enriched GO terms of DEGs.

Category	GO ID	Term	*N*	Expected	KS
BP	GO:0015766	Disaccharide transport	30	8.88	2.60*E* − 06
	GO:0007018	Microtubule-based movement	24	7.21	0.00034
	GO:0045886	Negative regulation of synaptic growth at neuromuscular junction	3	3.78	0.0004
	GO:0030239	Myofibril assembly	12	6.86	0.00056
	GO:0010998	Regulation of translational initiation by eIF2 alpha phosphorylation	2	0.97	0.0009

CC	GO:0016020	Membrane	269	228.58	5.90*E* − 05
	GO:0030286	Dynein complex	17	3.19	0.00024
	GO:0005819	Spindle	20	6.86	0.00052
	GO:0005879	Axonemal microtubule	7	1.13	0.00068
	GO:0045298	Tubulin complex	9	1.6	0.00093

MF	GO:0008011	Structural constituent of pupal chitin-based cuticle	1	4.39	5.80*E* − 15
	GO:0042302	Structural constituent of cuticle	17	14.32	1.10*E* − 07
	GO:0051119	Sugar transmembrane transporter activity	18	6.27	1.80*E* − 05
	GO:0003777	Microtubule motor activity	20	4.57	3.30*E* − 05
	GO:0005525	GTP binding	19	15.84	0.00026

BP: biological process; CC: cellular component; MF: molecular function; GO: Gene Ontology. *N* represents the number of DEGs enriched in this specific GO term. Expected: the expected value. KS value represents the significance of the GO term. Smaller KS value corresponded to more significance of the GO term.
